# The Role of Cytokines in Perioperative Neurocognitive Disorders: A Review in the Context of Anesthetic Care

**DOI:** 10.3390/biomedicines13020506

**Published:** 2025-02-18

**Authors:** Hyun Jung Koh, Jin Joo

**Affiliations:** Department of Anesthesiology and Pain Medicine, Seoul St. Mary’s Hospital, College of Medicine, The Catholic University of Korea, Seoul 06591, Republic of Korea; hjkoh92@naver.com

**Keywords:** perioperative neurocognitive disorder, postoperative delirium, postoperative cognitive dysfunction, blood–brain barrier, neuroinflammation, cytokine, anesthesia

## Abstract

Perioperative neurocognitive disorders (PNDs), including postoperative delirium, delayed neurocognitive recovery, and long-term postoperative neurocognitive disorders, present significant challenges for older patients undergoing surgery. Inflammation is a protective mechanism triggered in response to external pathogens or cellular damage. Historically, the central nervous system (CNS) was considered immunoprivileged due to the presence of the blood–brain barrier (BBB), which serves as a physical barrier preventing systemic inflammatory changes from influencing the CNS. However, aseptic surgical trauma is now recognized to induce localized inflammation at the surgical site, further exacerbated by the release of peripheral pro-inflammatory cytokines, which can compromise BBB integrity. This breakdown of the BBB facilitates the activation of microglia, initiating a cascade of neuroinflammatory responses that may contribute to the onset of PNDs. This review explores the mechanisms underlying neuroinflammation, with a particular focus on the pivotal role of cytokines in the pathogenesis of PNDs.

## 1. Introduction

The aging population presents significant challenges for patients undergoing surgery [1]. Perioperative neurocognitive disorders (PNDs), including postoperative delirium (POD), delayed neurocognitive recovery, and postoperative neurocognitive dysfunction, are particularly concerning for older surgical patients [2]. The intricate relationship between cytokines and cognitive dysfunction involves multiple mechanisms, such as blood–brain barrier (BBB) disruption, microglial activation, and oxidative stress. It is increasingly recognized that the immune system plays a pivotal role in influencing the central nervous system (CNS) after surgical trauma, potentially contributing to the development of PNDs [3]. However, the underlying mechanisms remain poorly understood.

One proposed explanation for PNDs is an exaggerated peripheral inflammatory response in surgical patients, which leads to the release of pro-inflammatory cytokines by macrophages. These cytokines may subsequently cross the BBB and activate microglial cells [4]. A comprehensive understanding of these processes is critical for developing effective strategies to predict, prevent, and treat POD and postoperative cognitive dysfunction (POCD).

In this review, we examine studies on cytokines related to PNDs and explore the role of anesthesia in modulating cytokine activity. A comprehensive search was conducted of the PubMed, PubMed Central, Medline, Google Scholar, and Google databases, using the keywords, “perioperative neurocognitive disorders”, “postoperative delirium”, “postoperative cognitive dysfunction”, “neuroinflammation”, “cytokine”, and “anesthesia.”

## 2. What Are PNDs?

### 2.1. Definition

A recent consensus [5] has recommended the term PNDs to describe cognitive changes occurring during the preoperative and postoperative periods. PNDs are further classified into preexisting cognitive impairment or delirium, delirium occurring within 7 days postoperatively, cognitive decline identified within 30 days postoperatively (referred to as delayed neurocognitive recovery), and cognitive decline detected between 30 days and 12 months postoperatively (postoperative neurocognitive dysfunction).

Patients with preexisting impairment in one or more cognitive domains—including complex attention, executive function, learning, memory, language, perceptual–motor, and/or social cognition—are considered to have baseline neurocognitive dysfunction. This dysfunction can be further classified as mild (mild cognitive impairment) or major (dementia) based on the severity of impairment.

POD is characterized by acute, fluctuating changes in attention, consciousness, and cognitive function. Although it may develop preoperatively, POD most frequently occurs within 7 days following surgery. Additional features of delirium may include psychomotor disturbances (hyperactive, hypoactive, or mixed), perceptual disturbances (e.g., hallucinations or delusions), emotional changes, and sleep–wake cycle disruptions, although these are not required for diagnosis.

**Figure 1:**
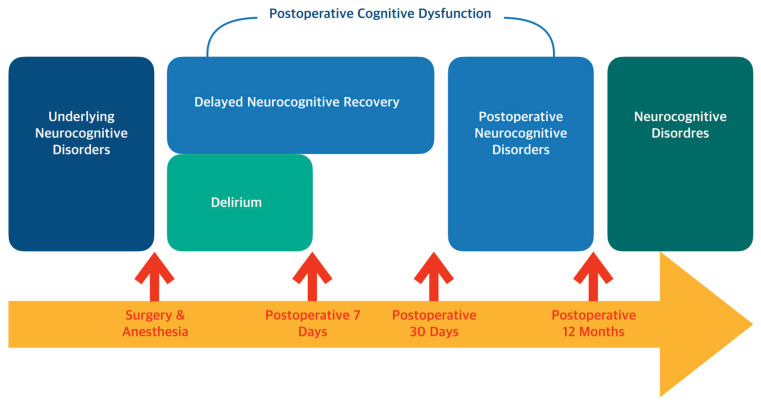
Definition of Postoperative Neurocognitive Disorders.

The term “delayed neurocognitive recovery” has replaced the traditional “early POCD” to reflect evidence that many patients recover fully from early cognitive impairments. This terminology highlights the potential for recovery. Meanwhile, “postoperative neurocognitive dysfunction” refers specifically to cognitive decline detected between 30 days and 12 months postoperatively. Beyond 12 months, the term “postoperative” is no longer applied to cognitive decline, as attributing causality to prior surgery and anesthesia becomes challenging. Generally, POCD includes both delayed neurocognitive recovery and postoperative neurocognitive dysfunction (Figure 1).

### 2.2. Incidence and Outcomes

In the general adult population, POD occurs in approximately 2.5–4.5% of cases. However, this incidence increases significantly to 12.0–23.8% among patients aged 60 years or older [6,7]. The risk of POD is influenced by the type and complexity of the surgical procedure. Data from the American College of Surgeons National Surgical Quality Improvement Program indicate that among 20,212 older patients, the incidence of POD varied by surgery type: 13.7% following cardiothoracic surgery, 13.0% after orthopedic or general surgery, 11.4% after vascular surgery, 8.0% following neurosurgery, 7.1% after plastic or otolaryngologic procedures, 6.6% following urological surgery, and 4.7% after gynecological procedures [6].

Similarly high rates of POD have been reported in other studies, including incidences of 15.3–23.4% following cardiovascular surgery [8,9], 16.9% after hip fracture surgery [10], and 22.7–26% following emergency surgery [11]. Additionally, POD was observed in 24.4% of patients admitted to the intensive care unit postoperatively [12].

The International Study of Post-Operative Cognitive Dysfunction, a landmark multicenter study, assessed cognitive decline in 1218 elderly patients undergoing major abdominal and orthopedic surgeries. Delayed neurocognitive recovery was identified in 25.8% of patients 1 week postoperatively, while POCD was diagnosed in 9.9% of the patients 3 months postoperatively [13]. Similarly, prospective studies involving adult patients undergoing non-cardiac surgeries reported a 30% incidence of delayed neurocognitive recovery at hospital discharge and a 10–13% incidence of POCD 3 months postoperatively [14]. A systematic review of 24 studies, including 8314 patients undergoing non-cardiac and non-neurological surgeries, estimated a pooled POCD incidence of 11.7% at 3 months postoperatively [15].

POD typically extends hospital stays by two to three days, increases the length of intensive care unit stays by two days, and raises the risk of readmission within 30 days. It is also strongly linked to cognitive decline, both shortly after surgery (one month) and over the long term [16]. Additionally, delirium is associated with a reduced quality of life related to health and an increased likelihood of developing dementia [2,17]. Delayed neurocognitive recovery is associated with adverse long-term effects. For instance, Kahl et al. [18] reported that individuals experiencing delayed recovery three to five days after surgery exhibited more significant cognitive impairments in memory, attention, action, and perception 12 months following a radical prostatectomy. Research also shows that patients with POCD three months post-surgery are twice as likely to encounter new challenges in daily activities compared to those without POCD and face higher risks of long-term mortality [19,20]. Although current studies indicate a significant relationship between POCD and long-term dementia [21], the evidence is still insufficient to draw definitive conclusions.

### 2.3. Assessment

Assessment of POD is typically conducted in patients at high risk or those experiencing any acute alteration in mental status. The DSM-5 is considered the gold standard for diagnosing POD. However, its application by non-psychiatric practitioners is often impractical. To date, more than 20 diagnostic tools have been developed and validated to aid in the diagnosis of delirium. Among these, commonly utilized screening tools include the Confusion Assessment Method (CAM), the Confusion Assessment Method for the Intensive Care Unit (CAM-ICU), the Brief Confusion Assessment Method (bCAM), the 3-Minute Diagnostic Interview for Delirium using the Confusion Assessment Method (3D-CAM), the Intensive Care Delirium Screening Checklist (ICDSC), and the 4As Test (4AT). Additionally, several instruments have been validated to assess the severity of delirium. These include the Memory Delirium Assessment Scale (MDAS), the Delirium Rating Scale-Revised-98 (DRS-R-98), and the Confusion Assessment Method-Severity (CAM-S), which are among the most frequently used tools for evaluating delirium severity [2,22].

A variety of neurocognitive tests are typically used to assess different cognitive domains, aiding in the detection of subtle and specific neurocognitive changes. Commonly employed tests include the digit span test with its forward and backward subtests, the Trail Making Test Part A, and the Digit Symbol Substitution Test. While conducting a full suite of these tests can be time-consuming, several streamlined instruments are employed to identify mild cognitive impairment. Recent research has explored POCD using straightforward tools such as the Abbreviated Mental Test and the Mini-Mental State Examination (MMSE), which, originally designed for dementia screening, lack the sensitivity needed to detect subtle cognitive declines. For more precise neuropsychometric assessment, sensitive tools like the Montreal Cognitive Assessment (MoCA), Addenbrooke’s Cognitive Examination III (ACE-III), and the Quick Mild Cognitive Impairment screen (Qmci) are available [2,14,23].

Currently, the primary biomarkers for predicting and diagnosing POCD encompass markers linked to immune inflammation, brain injury, brain protection, and biological rhythms, including amyloid β-protein (Aβ), tau protein, S100B, cortisol, and melatonin [24,25,26]. Various studies have sought to clarify the relationship between multiple cytokines and PNDs, with related findings to be discussed later in this review. Neuroimaging research has aimed to identify brain changes linked to POCD; a systematic review by Huang et al. [27] indicated that POCD might be associated with reduced volumes in the thalamus and hippocampus, existing white matter abnormalities, and decreased cerebral blood flow. Research continues to investigate biomarkers and neuroimaging as means to objectively diagnose PNDs, although the current evidence remains too limited to draw definitive conclusions.

## 3. PND Pathogenesis

Identified risk factors for POCD include advancing age, lower education level, and a history of cerebrovascular events without residual impairment. Potential predictors of early POCD include the duration of anesthesia, postoperative infections, subsequent surgeries, and respiratory complications [14]. Risk factors for POD largely overlap with those for POCD, including advanced age, low educational level, and frailty. POD is also influenced by the type of surgery and the anesthesia method employed [28].

The exact pathogenesis of PNDs remains elusive. Current studies investigate a range of factors, including genetics, epigenetics, neurotransmitters, brain injury, β-amyloid protein (Aβ) deposition, neuroinflammation, oxidative stress, the cholinergic anti-inflammatory pathway, synaptic dysfunction, and gut microbiota [4,26]. Cao et al. [29] have posited that individuals carrying the apolipoprotein E epsilon 4 allele (APOEε4)—a notable risk factor for Alzheimer’s disease (AD) marked by the loss of basal forebrain cholinergic neurons—may have an increased risk of developing POCD. Epigenetic mechanisms are believed to play a pivotal role in the development of POCD. Although the data show variability, they suggest that epigenetic alterations can affect neuroinflammation, neuronal development, and neurotransmitter secretion [30]. Recent studies in both human and animal models suggest that neuroinflammation, triggered by surgery or anesthesia, plays a significant role in PND onset and progression [26,28]. This review primarily focuses on neuroinflammation and the role of cytokines within this process.

### 3.1. Neuroinflammation

Inflammation is a protective response activated in reaction to external pathogens or damaged cells. For many years, the CNS was considered immunoprivileged due to the BBB, which acts as a physical barrier to prevent systemic inflammation from affecting the CNS. However, it is now increasingly recognized that the CNS is not entirely insulated from systemic inflammatory responses [31]. Similar to inflammation elsewhere in the body, neuroinflammation is a natural, protective physiological response in the brain designed to safeguard the CNS from harmful internal and external factors. While neuroinflammation serves a defensive role, excessive production of inflammatory mediators can have detrimental effects on the CNS [32].

Aseptic surgical trauma induces localized inflammation at the surgical site, which is further amplified by the release of peripheral pro-inflammatory cytokines [33,34]. These cytokines can compromise BBB integrity by upregulating cyclooxygenase-2 and matrix metalloproteinases, allowing pro-inflammatory cytokines to penetrate the CNS [34,35,36,37]. For instance, interleukin-1 (IL-1) and tumor necrosis factor-alpha (TNF-α) can stimulate cyclooxygenase-2 expression in neurovascular endothelial cells, promoting prostaglandin synthesis and further disrupting the BBB [36,37,38]. Pro-inflammatory cytokines, such as TNF-α, IL-1β, and IL-6, have been detected in hippocampal tissue in animal models and in human cerebrospinal fluid (CSF) following surgical trauma, indicating BBB breakdown [39,40,41,42].

Once the BBB is compromised, neuroinflammation is exacerbated by increased cytokine expression and microglial activation. Microglia, often referred to as the “resident macrophages” of the CNS, are essential for CNS maintenance, including synaptic pruning during development and synaptic scaling in neural plasticity [43]. Under normal conditions, microglia remain in an inactive state. However, inflammation and BBB disruption can activate microglia, leading to differentiation into two phenotypes: M1 (pro-inflammatory and potentially harmful) and M2 (anti-inflammatory and protective). The M1 phenotype is triggered by lipopolysaccharides (LPS) and the pro-inflammatory cytokine interferon-gamma, resulting in the production of oxidative metabolites, proteases, and cytokines, such as IL-1β, IL-6, and TNF-α. In contrast, the M2 phenotype is activated by anti-inflammatory cytokines, such as IL-4 and IL-13, promoting tissue repair and angiogenesis through the production of arginase-1 and anti-inflammatory cytokines, including IL-10 [43,44,45,46]. These dual roles of microglia highlight their critical influence on CNS homeostasis and response to inflammation. Once activated, microglia sustain and amplify neuroinflammation by increasing the production of pro-inflammatory cytokines [43]. Several factors, including trauma, aging, dementia, hypertension, stroke, depression, diabetes, and exposure to certain drugs and toxins, are known to contribute to neuroinflammation within the CNS [43,45,46].

Memory formation occurs in the hippocampus through a process known as long-term potentiation (LTP). While the mechanisms underlying the induction and maintenance of LTP at various synapses in the CNS are complex and remain a topic of debate, LTP is generally believed to occur via high-frequency glutamatergic activation of hippocampal neurons. Pro-inflammatory cytokines can disrupt neurotransmitter signaling in the hippocampus, leading to excitotoxic neuronal damage and cognitive impairment. This vulnerability is partly attributed to the high density of cytokine receptors in the hippocampus, making it particularly sensitive to elevated pro-inflammatory cytokine levels [4].

The role of neuroinflammation has been extensively studied in neurodegenerative diseases, such as AD and Parkinson’s disease (PD), but its involvement in PNDs has received comparatively less attention. Research on AD and PD has demonstrated the complexity of neuroinflammatory pathways contributing to brain pathology [45,46,47,48]. The neuroinflammation hypothesis of PNDs suggests that patients experience an exaggerated systemic inflammatory response to surgery. In this process, macrophages at the surgical site release excessive inflammatory mediators, such as cytokines, which subsequently trigger neuroinflammation in the CNS [34].

### 3.2. Cytokines

To date, approximately 200 different cytokines have been identified. These small proteins exhibit a broad range of biological functions and play a crucial role in regulating host responses to infection, immune reactions, inflammation, and trauma. While some cytokines exacerbate disease through pro-inflammatory actions, others mitigate inflammation and support healing as anti-inflammatory agents. Pro-inflammatory cytokines such as TNF-α, IL-1β, IL-6, and IL-8 play a crucial role in inducing fever, inflammation, and tissue damage, and in severe cases, can contribute to shock or even death. In contrast, anti-inflammatory cytokines like IL-4, IL-10, and IL-13 function as immunoregulatory molecules, helping to modulate the inflammatory response and maintain homeostasis, thereby preventing excessive tissue damage [49,50]. Cytokines, particularly IL-1β, TNF-α, and IL-6, are thought to interact with the brain via various pathways, including vagal afferents, crossing the BBB, or via the circumventricular regions. These peripheral cytokines trigger cytokine production by activated microglia, leading to a cycle of neuroinflammation.

### 3.3. Clinical Studies Investigating PNDs from the Perspective of Neuroinflammation and Cytokines

Several animal model studies have shown that cytokines contribute to neuroinflammation, which may lead to PNDs [41,42,51,52,53,54,55]. Building on these animal model studies, clinical research investigating the role of cytokines in neuroinflammation has been actively conducted over the past decade [39,56,57,58,59,60,61,62,63,64,65,66,67,68,69,70,71,72,73,74,75,76,77,78,79,80,81,82,83,84,85,86,87,88,89,90,91,92]. The most extensively studied cytokines include IL-6, IL-1β, TNF-α, high-mobility group box 1 protein (HMGB1), and S100B (Table 1).

IL-6 is a multifunctional cytokine that plays a crucial role in neuroinflammation and significantly impacts neuronal activity. In the central nervous system (CNS), it is either synthesized and released by neurons and glial cells or transported from peripheral sources. Under normal conditions, IL-6 levels in the CNS are relatively low, but they can rise dramatically in response to psychological stress, pathological conditions such as AD and PD, or stimulation by TNF-α. By promoting neuroinflammation, IL-6 is believed to contribute to the development and progression of PNDs, potentially exacerbating cognitive decline and other neurological impairments [93].

IL-1β is a versatile pro-inflammatory cytokine that plays a central role in coordinating inflammatory and host defense responses in peripheral tissues. In the healthy adult brain, IL-1β is expressed at low levels. However, following localized brain injury or insult, its expression is significantly upregulated by microglia [94].

TNF-α, another pro-inflammatory cytokine, is primarily produced by neurons and glial cells, including astrocytes and microglia, which are the predominant glial cell types. TNF-α plays a crucial role in regulating acute-phase inflammation by initiating signaling cascades of inflammatory cytokines, making it a central mediator of the inflammatory response [95].

**Table 1:** Clinical Studies on Perioperative Neurocognitive Disorders.

Study	Sample Size (*n*)	Cohort	Study Type	Surgical Procedure	CSF	Plasma
Plaschke K et al. [96]	114	>18	prospective, observational cohort study	open heart surgery		POST: IL-6 higher in POD; **implication**: inflammatory processes play a role in the development of POD
Hudetz JA et al. [97]	114	≥55	prospective, observational cohort study	open heart surgery		PRE–POST change: IL-6 higher in cognitive impairment; **implication**: significance of managing postoperative inflammation to improve cognitive outcomes
Li YC et al. [98]	37	>60	prospective, observational cohort study	hip surgery		PRE–POST change: IL-6, S100B higher in POCD; IL-1b, TNF-a (no significance); **implication**: significant relationship between elevated perioperative inflammatory responses with the incidence and severity of POCD
Ji MH et al. [99]	61	65–85	prospective, observational cohort study	hip surgery	PRE: IL-1β higher in POCD; IL-6 (no significance)	PRE–POST change: IL-1β, IL-6 (no significance); **implication**: the potential of these biomarkers as predictive tools for assessing the risk of POCD
Liu P et al. [100]	338	≥60	prospective, observational cohort study	major noncardiac surgery		PRE–POST change: IL-6 higher in POD; **implication**: IL-6 levels could be a valuable tool in the preoperative assessment to predict POD risk, potentially guiding preventive strategies.
Reinsfelt B et al. [101]	10	73 ± 6 yrs	prospective, observational cohort study	open heart surgery	PRE–POST change: IL-6, IL-8, TNF-a increased accompanied by increased AD-associated Aβ1–42; **implication**: increased risk of developing or exacerbating Alzheimer’s disease-related neurodegenerative processes after surgery	
Westhoff D et al. [102]	61	≥75	RCT	hip surgery	PRE: IL-1RA, IL-6 higher in POD; IP-10 higher in POD (no significance); IL-15 lower in POD (no significance); **implication**: the importance of preoperative evaluation of inflammatory markers in the CSF to anticipate and potentially mitigate the risk of POD	
Cape E et al. [56]	43	>60	prospective, observational cohort study	hip surgery	PRE: IL-1β, IL-1RA higher in POD; IFN-γ, IGF-1 (undetected); **implication**: IL-1β as a potential biomarker for POD risk	
Capri M et al. [57]	74	>65	case–control study	any kind of surgery		PRE: IL-6 higher in POD; IL-2 lower in POD (no significance); IL-8, IL-10 (no difference); **implication**: the importance of preoperative inflammatory status as a significant risk factor for adverse cognitive outcomes, particularly POD
Kazmierski J et al. [58]	113		prospective, observational cohort study	open heart surgery		POST: IL-2, TNF-α increased; **implication**: the association between systemic inflammation and POD
Lin GX et al. [59]	50	≥60	prospective, observational cohort study	major gastrointestinal surgery.		PRE–POST change: IL-6, HMGB1 higher in POCD; **implication**: HMGB1 as a predictor of cognitive decline enables early interventions and monitoring strategies to manage or mitigate POCD
Vasunilashorn SM et al. [60]	566	≥70	prospective, observational cohort study	major noncardiac surgery		PRE–POST change: IL-2, IL-6 higher in POD; **implication**: systemic inflammatory responses may play a key role in the onset of POD
Skrede K et al. [61]	19	≥65	prospective, observational cohort study	hip surgery		PRE–POST change: MCP-1 higher in POD; **implication:** the importance of MCP-1 as a potential biomarker for identifying patients at risk of POD
Zhang YH et al. [62]	63	≥65	prospective, observational cohort study	lumbar discectomy		PRE–POST change: IL-6, IL-10 higher in POCD; **implication**: elevated levels of these biomarkers are implicated in the development of POCD
Neerland ET et al. [63]	149	≥60	RCT	hip surgery	PRE: sIL-6R higher in POD (no significance)	PRE: IL-6, sIL-6R (no significance); **implication**: elevated levels of these inflammatory biomarkers in CSF can serve as potential predictors of delirium risk
Shen H et al. [64]	140	≥65	prospective, observational cohort study	open abdominal surgery		PRE: IGF-1 lower in POD; IL-6 increased (no significance); **implication**: utility of IGF-1 not only in predicting the occurrence of POD but also in understanding the underlying mechanisms
Hirsch J et al. [39]	10	≥55	prospective, observational cohort study	major knee surgery	PRE: IFN-α2 higher in POD and IFN-α2, IL-10 lower in POD; PRE–POST change: IL-6, IL-8, IL-10, MCP-1 higher in POD and IL-10 higher in POCD; **implication**: the inflammatory processes could potentially help in predicting postoperative cognitive complications	Delirium: PRE: MIP-1α, MIP-1β, IL-6 higher in POD increased and IL-4, IL-5, IL-6, IL-12, IFN-α2, IFN-γ lower in POCD; PRE–POST change: IFN-α2, IFN-γ, IL-4, IL-5, IL-12 lower in POD and IFN-α2, IL-12, and IL-4 lower in POCD
Kline R et al. [65]	30	≥65	prospective, observational cohort study	major noncardiac surgery		PRE–POST change: IL-6, IL-8, TNF-α higher in cognitive decline; IL-10 (no significance); **implication**: the potential impact of inflammation on POCD
Sun L et al. [66]	112	65–85	prospective, observational cohort study	oral cancer free flap surgery		PRE–POST change: IL-6 higher in POD; **implication**: elevated levels of these biomarkers may be linked to the onset of POD, indicating a possible inflammatory and neurochemical basis for postoperative cognitive impairments
Jiang J et al. [67]	44	≥60	prospective, observational cohort study	head and neck cancer		PRE: IGF-1 lower in POCD; PRE–POST change: TNF-α higher, IGF-1 lower in POCD; **implication**: higher TNF-α might inhibit the neuroprotective effects of IGF-I, contributing to POCD
Yu H et al. [68]	51	18–65	prospective, observational cohort study	cytoreductive surgery and hyperthermic intraperitoneal chemotherapy		PRE–POST change: IL-6, HMGB1,S100B higher in POCD; TNF-α (no significance); **implication**: the inflammatory response could be a contributing factor to the development of POCD
Guo HY [69]	51	60–82	prospective, observational cohort study	open heart surgery		POST: IL-1β, IL-6, MMP-3, MMP-9 higher in POCD; **implication**: these biomarkers could be crucial in understanding the pathophysiology of POCD
Sajjad MU et al. [70]	331	≥65	prospective, observational cohort study	hip surgery	PRE: IL-8 higher in POCD; TNF-α, IL-1β (undetected); **implication**: IL-8 could be involved in the inflammatory processes associated with cognitive disorders and might serve as a biomarker for neuroinflammatory conditions	
Chen Y et al. [71]	266	≥18	prospective, observational cohort study	open heart surgery		PRE–POST change: IL-6 higher in POD; **implication**: elevated IL-6 as a predictive biomarker for POD
Lin X et al. [72]	447	≥65	prospective, observational cohort study	hip/knee surgery	PRE: IL-6, TNF-α higher in POD; **implication**: specific markers in the cerebrospinal fluid are significantly linked to the development of POD	PRE–POST change: IL-6, TNF-α higher in POD
Yuan Y et al. [73]	34	≥65	prospective observational case–control preliminary study	hip surgery		PRE–POST change: IL-6 higher in POD; IL-1β, TNF-α (no significance); **implication**: elevated levels of these biomarkers in plasma exosomes may correlate with a higher risk of developing POD
Danielson M et al. [74]	27	65–76	prospective, observational cohort study	hip/knee surgery	PRE–POST change: IL-6, IL-8 higher in POCD (transiently increased CSF/serum albumin ratio & CSF, serumS100B); **implication**: neuroinflammation could play a significant role in the cognitive deterioration	
Casey CP et al. [75]	114		prospective, observational cohort study	major noncardiac surgery		PRE–POST change: IL-8 higher in POD (in relation with severity); IL-1β, IL-1RA, IL-2, IL-4, IL-6, IL-10, IL-12p70, MCP-1, TNF-α (no significance); **implication**: the presence of higher neurofilament light levels and these biomarkers indicates neuronal damage or neuroaxonal dysfunction, which could be contributing to the development of POD
Kavrut Ozturk N et al. [76]	82	>50	prospective, observational cohort study	Robotic-Assisted Laparoscopic Radical Prostatectomy		PRE–POST change: S100B higher in POCD; **implication**: S100B could serve as a biomarker for detecting POCD
Ballweg T et al. [77]	110		prospective, observational cohort study	open thoracoabdominal aortic aneurysm/TEVAR		PRE–POST change: IL-8, IL-10 higher in POD; IL-1β, IL-1RA, IL-2 (no significance); **implication**: tau protein and these biomarkers could potentially be used as a biomarker for predicting and monitoring POD, allowing for targeted interventions to prevent or mitigate POD
CheheiliSobbi S et al. [78]	89	≥50	prospective, observational cohort study	open heart surgery		PRE–POST change (ex vivo-stimulated production): TNF-α, IL-6, IL-10 higher in POD (no significance); **implication**: significant link between the degree of immunoparalysis and the likelihood of developing POD
Lv XC et al. [79]	221	>18	retrospective study	open heart surgery		PRE–POST change: IL-6 higher in POD; **implication**: IL-6 could serve as a useful biomarker for identifying patients at higher risk of POD
Zhang S et al. [80]	390	>18	prospective, observational cohort study	open heart surgery		PRE–POST change: IL-6 higher in POD; **implication**: IL-6 as a valuable biomarker for better managing and potentially preventing POD
Taylor J et al. [81]	72	≥65 yrs	prospective, observational cohort study	non-intracranial surgery	IL-6 correlates with S100B; **implication**: the integrity of the BBB and the inflammatory response may play significant roles in the development of POD	PRE–POST change: S100B higher in POD
Wu JG et al. [82]	64	≥21 yrs	prospective, observational cohort study	non-intracranial surgery	PRE–POST change: IL-18 (no significance)	PRE–POST change: IL-18 (no significance); **implication**: IL-18 may be involved in the neuroinflammatory pathways that contribute to POD
Khan SH et al. [83]	71	≥18 yrs	secondary analysis of blood samples from a RCT	esophagectomy		PRE–POST change: IL-8, IL-10 higher in POD; S100B increase and IGF-1 (correlated with greater POD severity); **implication**: a possible association between systemic inflammation, neuroinflammation, and the pathogenesis of POD
Oren RL et al. [84]	76	45–60 or ≥70 yrs	prospective observational study	spine surgery		PRE–POST change: IL-8, IL-6 higher in POD; **implication**: older patients exhibit distinct cytokine alterations that may contribute to increased POD
Zhang Y et al. [85]	126	≥60 yrs	prospective, observational cohort study	hip/knee surgery		PRE–POST change: IL-6, sIL-6R higher in POD; IL-1β, IL-2, IL-4, IL-10, TNF-α (no significance); **implication**: monitoring cytokine profiles pre- and postoperatively could aid in risk stratification and early intervention of POD
Su LJ et al. [86]	318	≥18 yrs	prospective, observational cohort study	open heart surgery		Post: IL-6, TNF-α, sTNFR-1, sTNFR-2 higher in POD; IL-1β (no significance); **implication**: targeting inflammation and neuroinflammatory pathways could be a promising approach for reducing the incidence and severity of POD
Imai T et al. [87]	221	24–88 yrs	retrospective study	head and neck cancer		POST: IL-6 higher in POD; **implication**: IL-6 as a potential biomarker for early identification of patients at risk for hyperactive POD
Taylor J et al. [88]	170	≥65 yrs	prospective, observational cohort study	non-intracranial surgery		POST: IL-6 higher in cognitive decline; **implication**: monitoring IL-6 levels postoperatively could serve as a biomarker for predicting cognitive recovery
Ruhnau J et al. [89]	44	≥60 yrs	prospective, observational cohort study	spine surgery		PRE–POST change: S100B, IL-6, IL-1β increased in POD; **implication**: pro-neuroinflammatory state before surgery may predispose patients to POD
Lozano-Vicario L et al. [90]	60	≥75 yrs	prospective, observational cohort study	hip surgery	PRE: CXCL9 lower in POD	PRE: CXCL9 lower in POD; **implication**: proteomic profiling of serum and CSF can help identify biomarkers linked to POD risk
Zhang S et al. [91]	212	≥18 yrs	prospective, observational cohort study	open heart surgery		POST: IL-6 higher in POCD; **implication**: systemic inflammation, particularly IL-6, is a critical factor in both POD and POCD development
Ko H et al. [92]	43	>30 yrs	prospective, observational cohort study	open heart surgery		PRE: TNF-α lower in POD; IL-6 higher (No significance), IL-1β (no difference); **implication**: strong evidence linking postoperative immune dysregulation with the onset of POD

BBB, blood–brain barrier; CSF, cerebrospinal fluid; CXCL 9, C-X-C motif chemokine ligand 9; HMGB1, high-mobility group box 1 protein; IGF-1, insulin-like growth factor-1 IL, interleukin; IL-1RA, interleukin-1 receptor antagonist; IP-10, interferon gamma-induced protein 10; IFN-γ, interferon gamma; MCP-1, monocyte chemoattractant protein-1; MIP-1α, macrophage inflammatory protein-1 alpha; S100B, S100 calcium binding protein B; sIL-6R, soluble interleukin-6 receptors; sTNFR, soluble tumor necrosis factor receptors; TNF-α, tumor necrosis factor alpha; PRE, preoperative; POST, postoperative; PRE–POST change, preoperative–postoperative difference; POCD, postoperative cognitive dysfunction; POD, postoperative delirium; RCT, randomized controlled trial.

HMGB1 proteins are part of the damage-associated molecular pattern family. These highly conserved non-histone nuclear proteins play a crucial role in maintaining chromatin DNA structure. HMGB1 is involved in driving pathogenic inflammatory responses and has been implicated in a variety of conditions, including epilepsy, septic shock, ischemia, PD, and AD [103]. Excessive activation of IL-1β and TNF-α receptors disrupts neuronal function by downregulating metabotropic glutamate receptors, which in turn enhances AMPA/NMDA signaling and interferes with LTP. Additionally, HMGB1 can amplify glutamate signaling through NMDA receptors, increasing the influx of glutamate into hippocampal neurons and ultimately causing glutamate toxicity. TNF-α further exacerbates this process by suppressing inhibitory neurotransmission through the downregulation of GABA receptors, disrupting the delicate balance between excitatory and inhibitory signaling, and promoting glutamate toxicity. This harmful cycle is intensified by T-cell-mediated glutamate release from activated microglia via a distinct glutamate transporter subtype. Together, these mechanisms contribute to glutamate toxicity in the hippocampus, leading to neuronal death and cognitive dysfunction [4,104].

Research into the relationship between plasma or serum cytokine levels and PNDs has yielded inconclusive results. Chen et al. [71] reported that IL-6 levels significantly increased postoperatively compared to preoperative levels in patients who developed POD following cardiac surgery. Similarly, Lin et al. [72] observed a significant postoperative increase in IL-6 levels in patients with POD in a study of 447 individuals undergoing knee or hip surgery. In contrast, Cheheili-Sobbi et al. [78] found no significant postoperative increase in IL-6 levels in patients with POD after cardiac surgery. Additionally, Casey et al. [75] reported no significant correlation between IL-6 levels and the occurrence of POD before and after non-cardiac surgery. Similarly, Kline et al. [65] observed an increase in TNF-α levels postoperatively compared to preoperative levels in patients who developed POCD. However, Yu et al. [68] found that this change was not statistically significant. The authors suggested that these inconsistencies could be attributed to variations in cytokine analysis methods, the timing of sample collection, differences in patient group composition, or the combination of prevalent and incident delirium cases in some studies.

Examining cytokines in CSF rather than serum or plasma offers a more rational approach when studying PNDs. Since CSF is in direct contact with the brain’s extracellular fluid, it provides a more accurate indicator of central biochemical changes compared to peripheral blood markers. Several cytokines have been found to be increased in the CSF following surgery, with IL-6 and IL-8 showing more significant elevations in the CSF compared to serum [39,56,63,70,72,74,101,102]. These findings suggest that the interactions between peripheral and central cytokines could reflect impaired BBB function, a potential mechanism underlying PNDs. Studies by Ji et al. [99], Neerland et al. [63], and Lin et al. [72] have demonstrated consistent changes in cytokine levels between CSF and plasma, supporting the hypothesis that PNDs are caused by neuroinflammation resulting from BBB disruption.

S100B, also known as S100 calcium-binding protein B, is primarily produced by astrocytes in brain tissue. Elevated serum or urine levels of S100B are often interpreted as being a result of increased CSF levels, which may arise due to astroglial activation or disruption of the BBB [105]. Taylor et al. [81] conducted a study to investigate neuroinflammation caused by BBB disruption as a mechanism underlying POD. That study, conducted on 72 patients aged 65 years and older who underwent non-intracranial surgery, demonstrated that changes in S100B, a plasma biomarker of astrocytic injury/activation, were associated with the incidence and severity of delirium. Importantly, these changes were consistent with alterations in CSF IL-6 levels. Several studies have also shown elevated S100B levels in the blood of PND patients, indicating a close relationship between PNDs, BBB disruption, and neuroinflammation [68,76,83,89,98].

### 3.4. Therapeutic Approaches for PNDs Based on Pathogenesis

As previously mentioned, various mechanisms of PNDs, including the neuroinflammatory pathway, have been proposed, but the exact mechanisms remain unclear. Consequently, a variety of preventive and treatment approaches are currently being suggested. These treatments are generally categorized into the following approaches: inhibiting inflammatory mediators to block inflammation, supporting and enhancing neuronal health before and during surgery such as prehabilitation, modulating gut microbiota, or addressing neurotransmitters such as cholinergic transmitters. Through these management strategies, many patients experiencing early cognitive decline eventually recover, although approximately one-sixth to one-half still exhibit symptoms of POCD during the follow-up period [13,106].

Considering the significant link between neuroinflammation and PNDs, focusing on inflammatory pathways offers a promising approach for therapy. Both preclinical and clinical research indicates that nonsteroidal anti-inflammatory drugs (NSAIDs) including cyclooxygenase-2 inhibitors and corticosteroids may mitigate neuroinflammatory responses by lowering the levels of pro-inflammatory cytokines, including IL-6 and TNF-α [107]. Inhibitors of TNF-α and the IL-6 receptor, such as infliximab and tocilizumab, have shown neuroprotective effects in animal studies and could potentially decrease the incidence of PNDs [108]. Dietary polyphenols like resveratrol and curcumin, known for their anti-inflammatory and antioxidant capabilities, have been found to reduce neuroinflammation and are known for their potential use as therapeutic agents in preventing or ameliorating cognitive decline [109]. Some anesthetic methods are known to reduce PNDs by modulating neuroinflammation. Inhalation anesthesia, compared to total intravenous anesthesia (TIVA) using propofol, may cause greater damage to the nervous system, with delayed recovery of cognitive function [110]. Dexmedetomidine (DEX), an α2-adrenergic agonist, has demonstrated neuroprotective properties by diminishing neuroinflammation and lowering the incidence of POD in elderly patients [111]. The impact of anesthesia methods on PNDs will be discussed in more depth later in this paper. Omega-3 polyun-saturated fatty acids are renowned for their anti-inflammatory properties and are consequently linked to improved cognitive resilience [112].

Optimizing perioperative neuronal health can bolster cognitive resilience and aid in preventing PNDs. Engaging in brain prehabilitation can enhance neural plasticity and build cognitive reserve, potentially reducing the severity of PNDs [113]. Additionally, regulating sleep with melatonin has been shown to offer neuroprotective benefits by pre-serving circadian rhythms and minimizing oxidative stress [114]. Furthermore, avoiding hypoglycemia during the perioperative period can reduce neuroinflammation and improve postoperative cognitive outcomes [115].

The gut microbiota, residing within the intestinal tract, can interact with the brain bidirectionally through the microbiota–gut–brain axis. This communication allows the gut microbiota to affect the cognitive functions and behaviors of the host [26]. Modulating the composition of gut microbiota through the intake of dietary live microbes and nondietary probiotic/prebiotic could lessen systemic inflammation and enhance cognitive function [116]. These lipids, known for their anti-inflammatory effects, have been linked to enhanced cognitive resilience.

Various pharmacological agents have been explored for the prevention and treatment of PNDs. Cholinesterase inhibitors, such as donepezil, have shown potential in reducing the risk of PNDs by enhancing cholinergic neurotransmission [117]. Additionally, statins, known primarily for their lipid-lowering effects, may also offer neuroprotective benefits by modulating neuroinflammatory pathways [118]. Despite the growing interest in managing PNDs, effective strategies are still limited. Nonetheless, the preventive and treatment methods mentioned above hold promise in decreasing the incidence and severity of PNDs. Future research should concentrate on validating these approaches through large-scale clinical trials and developing tailored interventions for high-risk patients.

## 4. Influence of Anesthesia on PNDs

Growing evidence suggests that PNDs arise from the combined effects of surgical procedures and anesthesia. Although some studies have indicated that anesthesia alone may not directly cause cognitive changes, anesthetic agents have been shown to influence glial cell phenotypes and modulate their activation, potentially leading to either beneficial or harmful effects on the CNS [119].

Neuroinflammatory responses caused by surgical insult are unavoidable for patients, particularly as the aging population grows. PNDs have emerged as a significant postoperative complication, prompting ongoing efforts to mitigate their occurrence. The relationship between the use of specific anesthetic agents and PNDs has been widely studied, though the findings are sometimes inconsistent. Generally, it is estimated that the shorter the duration of action of an anesthetic agent, the shorter the duration of POCD in the immediate postoperative period [30]. Various studies have investigated the impact of anesthetic methods on PNDs through different approaches. Table 2 summarizes research that has identified how anesthetic methods influence PNDs by affecting neuroinflammation mechanisms through cytokine modulation.

### 4.1. Impact of Anesthetic Method on PNDs from the Perspective of Neuroinflammation and Cytokines

The most common general anesthetics include halothane-based inhalation agents and TIVA, with propofol being the most widely used. However, the impact of TIVA and volatile anesthetics on PNDs in humans remains uncertain. While numerous studies have explored this topic, many have been limited by small sample sizes, suboptimal diagnostic methodologies, or an insufficient focus on this specific question.

Li et al. [120] conducted a randomized controlled study on 544 patients aged 60 years and older undergoing laparoscopic abdominal surgery to compare the effects of sevoflurane and propofol on POCD. The study revealed no significant difference in the incidence of delayed neurocognitive recovery between patients receiving propofol-based anesthesia and those receiving sevoflurane-based anesthesia. This suggested that the choice of anesthetic may not be a modifiable factor for preventing delayed neurocognitive recovery. However, the study identified elevated serum IL-6 levels as an independent risk factor for delayed neurocognitive recovery, providing clinical evidence of inflammation’s role in its development.

In another study, Qiao et al. [121] examined 90 patients aged 65–75 years undergoing esophageal cancer surgery to assess the incidence of POCD and changes in cytokine levels. Patients were anesthetized with sevoflurane, propofol, or sevoflurane, along with pre-treatment with methylprednisolone. The study found a higher incidence of POCD in patients receiving sevoflurane anesthesia compared to those on a propofol regimen. Methylprednisolone pre-treatment reduced the incidence of POCD in elderly patients undergoing sevoflurane anesthesia. The authors concluded that methylprednisolone could mitigate POCD by suppressing IL-6 and TNF-α levels.

Studies have also compared regional anesthesia (RA) with general anesthesia, or RA combined with general anesthesia (via inhalation or intravenous agents), as well as different RA techniques [122,123,124,125,126]. Local anesthetics, a cornerstone of RA, possess anti-inflammatory properties. These include interrupting nociceptive transmission to reduce neurogenic inflammation and exerting intrinsic anti-inflammatory effects independent of sodium channel blockade, acting directly on immune cells. Unlike immunosuppressive drugs, local anesthetics modulate excessive inflammatory responses without compromising the body’s natural defenses. RA’s modulation of perioperative inflammation is also linked to its opioid-sparing effect, which reduces exposure to opioids, known for both their immunosuppressive and pro-inflammatory properties [127].

**Table 2:** Clinical Studies on Perioperative Neurocognitive disorders and Anesthetic Methods.

Study	Sample Size (*n*)	Cohort (yrs)	Study Type	Surgical Procedure	Anesthetic Exposure	Key Findings
Lili X et al. [128]	40	>65	RCT	elective abdominal surgery	UTI vs. control	lower incidence of POCD in the UTI group; S100B, IL-6 decreased in the UTI group; **implication**: UTI may offer neuroprotective benefits by mitigating inflammation and oxidative stress, thereby improving early POCD
Jildenstål PK et al. [129]	450		RCT	elective eye surgery	AAI vs. control	IL-6 lower in the AAI group; IL-6 higher in patients with a MMSE < 25; **implication**: individualized anesthesia depth monitoring may play a protective role against postoperative neuroinflammation and cognitive dysfunction
Li Y et al. [130]	120	>60	RCT	laparoscopic cholecystectomy	DEX vs. control	IL-1β, IL-6 lower in the DEX group; IL-1β, IL-6 higher in patients who developed POCD on day 1 following surgery; **implication**: DEX may enhance early POCD and modulate inflammatory response
Jia Y et al. [131]	240	≥70	RCT	open colorectal surgery	fast-track surgery vs. traditional	POD occurrence lower in the fast-track surgery group; IL-6 decreased in fast-track surgery group; **implication**: the implementation of fast-track surgery protocols in elderly surgical populations to enhance recovery and reduce POD
Chen K et al. [132]	87	>65	RCT	spine surgery	lidocaine vs. control	MMSE scores markedly higher in the lidocaine group; S100B, IL-6 decreased in the lidocaine group; **implication**: lidocaine infusion shows promise as a neuroprotective strategy for preventing early POCD
Qiao Y et al. [121]	90	65–75	RCT	resection of an esophageal carcinoma	Sevo vs. Sevo-PRE methylprednisolone vs. TIVA	MMSE, MoCA scores lower in the Sevo group; TNF-α, IL-6, S100B higher in the Sevo group > IV propofol group > Sevo + PRE methylprednisolone; **implication**: TIVA with propofol appears to be a safer alternative for minimizing POCD
Chen W et al. [133]	148	61–89	retrospective study,		DEX vs. control	incidence of POCD lower in the DEX group; IL-6, TNF-α lower in the DEX group; **implication**: DEX may be an effective agent in reducing POCD and neuroinflammation
Wang KY et al. [134]	80	≥60	RCT	radical resection for esophageal cancer under one lung ventilation	UTI vs. control	incidence of POCD lower in the UTI group; S100B, IL-6 lower, IL-10 higher in the UTI group; **implication**: UTI may be a promising therapeutic option for reducing POCD
Xin X et al. [135]	120	>65	RCT	hip arthroplasty	PRE hypertonic saline vs. normal saline	lower risk of POD in the PRE hypertonic group; higher TNF-α associated with POD, IL-1β, IL-6, IL-10, S100B not significantly related to POD; **implication**: hypertonic saline, with its hemodynamic stability and anti-inflammatory effects, may be a promising intervention for reducing the risk of POD
He Z et al. [136]	90	65–75	RCT	laparotomy colon carcinoma surgery	ischemic preconditioning vs. control	MoCA scores higher in theischemic preconditioning group; IL-1β, TNF-α, S100B lower in the remote ischemic preconditioning group; **implication**: ischemic preconditioning can improve early postoperative cognitive function potentially through the inhibition of inflammatory responses
Lu XY et al. [137]	70		RCT	radical surgery for cervical cancer	remifentanil vs. fentanyl	POCD occurrence lower in the remifentanil group; IL-6, TNF-α lower in the remifentanil group; **implication**: remifentanil is effective in promoting faster recovery and better cognitive outcomes
Zhang H et al. [138]	120	65–75	RCT	esophageal carcinoma resection	midazolam + propofol vs. midazolam + Sevo vs. DEX + propofol vs. DEX+ Sevo	MMSE and MoCA scores lower in the midazolam + Sevo (vs midazolam + propofol), MMSE and MoCA scores higher in the DEX + Sevo (vs midazolam); IL-6, TNF-α, S100B higher in the midazolam + Sevo (vs midazolam + propofol), IL-6, TNF-α, S100B lower in the DEX + Sevo (vs midazolam); POCD incidence higher in Sevo anesthesia; **implication**: DEX is an effective adjunctive therapy for mitigating POCD by reducing inflammation and oxidative stress
Lee C et al. [139]	354	>65	RCT	laparoscopic major non-cardiac surgery	intraoperative DEX infusion vs. intraoperative DEX bolus vs. control	POCD incidence and duration lower in DEX infusion, POCD duration lower in DEX bolus (vs control); IL-6 lower in DEX groups; **implication**: the timing and dose of DEX are crucial in preventing POD, with continuous infusion showing the most significant benefits
Quan C et al. [140]	120	≥60	RCT	abdominal surgery	Deep (BIS target 30–45) vs. Light (BIS target 45–60)	POCD incidence lower in the Deep group (at 7 days after surgery); IL-1β lower in the Deep group, IL-10, S100B no significance; **implication**: BIS-guided deep anesthesia can reduce short-term POCD and peripheral inflammation
Kim JA et al. [141]	143	18–75	RCT	thoracoscopic lung resection	DEX-Sevo vs. Sevo	emergence agitation lower in Dex-Sevo group, POD no difference; IL-8, IL-10 lower & IL6/IL10 ratio, IL8/IL10 ratio in Dex-Sevo group, IL-6 no significance; **implication**: DEX reduces emergence agitation but does not significantly impact POD
Wang Y et al. [142]	100	20–60	RCT	thoracotomy for esophageal cancer	intercostal nerve block vs. control	MMSE score higher in the intercostal nerve block group; IL-6, TNF-α lower, IL-10 higher in the intercostal nerve block group; **implication**: intercostal nerve block improves early POCD and reduces inflammation
Hassan WF et al. [143]	80		RCT	laparoscopic cholecystectomy	magnesium sulphate vs. control	POST S100B higher in the control group (vs PRE), no difference in the magnesium sulphate group; **implication**: magnesium sulphate during conventional general anesthesia can protect against POCD and attenuate the postoperative elevation of serum S100B
Xin X et al. [144]	60	>65	RCT	laparoscopic cholecystectomy	DEX vs. control	POD incidence lower in DEX group; TNF-α lower, IL-10 higher in Dex group; **implication**: DEX did not significantly reduce the incidence of POD, but it may have potential benefits in reducing neuroinflammation
Mei B et al. [145]	366	≥65	RCT	total knee arthroplasty	spinal anesthesia supplemented with propofol vs. Dex	POD incidence lower in DEX group; S100B higher in the propofol group, TNF-α lower, IL-6 no difference; **implication**: the cognitive benefits of DEX are not related to its modulation of peripheral inflammation
Uysal Aİ et al. [125]	114	>65	RCT	trochanteric femur fracture surgery	spinal anesthesia supplemented with paracetamol vs. femoral nerve block	POD incidence lower in the femoral nerve block group; IL-8 lower in the femoral nerve block group, IL-6 no significance; **implication**: the incidence of POD was lower in the femoral nerve block group, but not statistically significant
Wang J et al. [146]	71	≥65	RCT	prone spinal surgery	lung-protective ventilation vs. conventional mechanical ventilation	POD incidence lower in the lung protective ventilation group; IL-6 lower, IL-10 higher in the lung protective ventilation group; **implication**: lung-protective ventilation may reduce POD by inhibiting inflammation and improving cerebral oxygen metabolism
Hu J et al. [147]	177	60–80	RCT	transthoracic oesophagectomy	TIVA vs. TIVA-DEX	POD incidence, emergence agitation lower in TIVA-DEX group; IL-6 lower TIVA-DEX group; **implication**: adding DEX to TIVA safely reduces POD and emergence agitation with associated reductions in IL-6 levels
Oh CS et al. [148]	82	>50	RCT	total hip replacement	moderate vs. deep NMB	POD no difference; IL-6 lower in the deep NMB group; **implication**: deep NMB may reduce inflammation, but its impact on the incidence of POD is not statistically significant
Jiang P et al. [126]	142	18–80	RCT	esophagectomy	general anesthesia vs. general anesthesia combined with epidural anesthesia	MoCA score higher in the general anesthesia combined with epidural anesthesia group; IL-6, IL-8, TNF-α lower in the general anesthesia combined with epidural anesthesia group; **implication**: combining general and epidural anesthesia may reduce the incidence of POCD by suppressing the inflammatory response
Li Y et al. [120]	544	≥60	RCT	laparoscopic abdominal surgery	Sevo vs. propofol	POCD no difference; associated with an increased likelihood of delayed neurocognitive recovery; IL-6 higher in POCD; **implication**: the choice between propofol and sevoflurane does not significantly affect the incidence of delayed neurocognitive recovery, but high IL-6 levels may be an independent risk factor for delayed neurocognitive recovery
Zhang Z et al. [149]	174	18–79	RCT		DEX vs. control	POCD incidence lower in DEX group; TNF-α, IL-6 lower in DEX group; **implication**: DEX has a protective effect on cognitive function and reduces inflammation
Huang Q et al. [150]	90	≥60	RCT	laparoscopic radical gastrointestinal tumor resections	insulin (20 U/0.5 mL insulin administered intranasally) vs. control	POD incidence lower in the insulin group; TNF-α, IL-6, IL-1β lower in the insulin group; **implication**: repeated preoperative intranasal administration of insulin effectively reduces the incidence of POD and lowers serum pro-inflammatory markers
Feng T et al. [122]	60	18–80	RCT	total hip arthroplasty	inside approach of the fascia iliaca compartment block (FICB) vs. outside approach of the FICB	POCD incidence lower in the inside approach group; IL-1b, IL-6 lower in the inside approach group; **implication**: inside approach of FICB provides lower incidence of POCD, and reduced serum cytokine levels
Chen S et al. [123]	103	>65	RCT	cardiac surgery	transversus thoracis muscle plane (TTMP) block vs. control	POCD lower in TTMP block group; IL-6,TNF-α,S-100β lower in TTMP block group; **implication**: TTMP blocks can improve postoperative cognitive function and reduce inflammation
Wang W et al. [151]	100	60–85	RCT		DEX vs. control	MMSE and MoCA scores higher in DEX group; IL-6,TNF-α,S-100B lower in DEX group; **implication**: DEX anesthesia alleviates early POCD and reduces inflammation
Wang JY et al. [152]	159	65–85	RCT	thoracoscopic lobectomy	goal-directed therapy vs. conventional	POD incidence lower in the goal-directed therapy group; IL-1β, IL-6, TNF-α, S-100B lower in the goal-directed therapy group; **implication**: goal-directed therapy can reduce perioperative inflammatory factor levels and the incidence of POD
Tang Y et al. [153]	120	60–80	RCT	hepatic lobectomy	0.3 μg, 0.6 μg/kg/hr DEX vs. control	POD & POCD incidence lower in 0.3 μg, 0.6 μg/kg/hr DEX group; TNF-α, IL-1β lower, IL-10 higher in 0.3 μg, 0.6 μg/kg/hr DEX group; **implication**: DEX effectively reduces the incidence of POD and POCD by modulating the balance between pro-inflammatory and anti-inflammatory responses
Xiang XB et al. [154]	168	65–80	RCT	laparoscopic gastrointestinal surgery	methylprednisolone vs. control	POD incidence lower in the methylprednisolone group; TNF-α loer in the methylprednisolone group; methylprednisolone does not reduce the severity of POD; **implication**: methylprednisolone reduces the incidence of POD potentially by reducing circulating markers of endothelial degradation
Kurup MT et al. [155]	64	60–80	RCT	open abdominal surgery	DEX vs. lidocaine	TNF-α, IL-6, S100B levels (no significance), IL-1 decreased in DEX (vs lidocaine), POCD no significance; **implication**: DEX and lidocaine are effective in reducing postoperative cognitive decline, with DEXshowing a slight advantage
Lai Y et al. [156]	90	>65	RCT	thoracoscopic lobectomy or segmentectomy	DEX vs. lidocaine vs. control	POD incidence no significance; IL-6, TNF-α lower in DEX and lidocaine; **implication**: DEX and lidocaine reduces surgical stress and inflammatory responses, although it does not significantly impact the incidence of POD
Xu F et al. [157]	84	≥60	RCT	shoulder arthroscopy	PRE hypertonic saline vs. normal saline	POD lower in the PRE hypertonic saline group; IL-6, TNF-α lower in the PRE hypertonic saline group; **implication**: preoperative hypertonic saline can reduce the incidence of POD and the immune-inflammatory response
Han C et al. [158]	84	≥60	RCT	gastrointestinal surgery	esketamine vs. control	incidence of delayed neurocognitive recovery lower in the esketamine group, no difference in POCD at 3 months after surgery; IL-6 and S100B lower in the esketamine group; **implication**: subanesthetic doses of esketamine may reduce the incidence of delayed neurocognitive recovery and improve early postoperative cognitive function
Zhu M et al. [124]	60	>65	RCT	hip surgery	quadratus lumborum block vs. control	POCD incidence lower in the quadratus lumborum block group; HMGB1, IL-6 lower in the quadratus lumborum block group; **implication**: quadratus lumborum block improves postoperative cognitive function and reduces inflammation
Zhi Y et al. [159]	140	60–85	RCT		TIVA vs. TIVA-etomidate	MMSE, MoCA scores higher in TIVA-etomidate group; IL-6, S100B lower, IL-10 higher in TIVA-etomidate group; **implication**: combination of etomidate and propofol for TIVA can alleviate POCD and reduce inflammation and stress response
Mi Y et al. [160]	116	≥60	RCT	noncardiac surgery	insulin (40 U/1 mL administered intranasally) vs. control	POCD incidence lower in the insulin group; TNF-α lower in the insulin group; **implication**: intranasal insulin administration reduces the incidence of POCD and alleviates peripheral inflammatory levels
Sun M et al. [161]	140		RCT	orthopaedic surgery or pancreatic surgery	insulin (40 IU/400 μL administered intranasally) vs. control	MMSE and MoCA-B scores higher, POD incidence lower in the insulin group; IL-6, S100B lower in the insulin group; **implication**: intranasal insulin administration may improve postoperative cognitive function and reduce the incidence of POD
Fu W et al. [162]	132	60–80	RCT	ureteroscopic holmium laser lithotripsy	etomidate-remifentanil-DEX vs. control	MMSE scores higher in the etomidate-remifentanil-DEX group; S100B lower in the etomidate-remifentanil-Dex group; **implication**: combination of DEX and etomidate improves postoperative cognitive function and attenuates the plasma concentrations of S100B
Luo T et al. [163]	129		RCT	non-cardiac thoracic surgery	0.2, 0.5 mg/kg esketamine vs. control	MMSE scores no difference; IL-6, S100B lower in the esketamine groups; **implication**: esketamine may help reduce postoperative negative emotions and early cognitive disorders by modulating the inflammatory response
Hsiung PY et al. [164]	110	>20	RCT	thoracoscopic tumor resection	nonintubated vs. intubated	Qmci-TW higher in the nonintubated group; IL-6 lower in the nonintubated group; **implication**: nonintubated thoracoscopic surgery is associated with improved postoperative neurocognitive recovery and reduced perioperative inflammation
Yin WY et al. [165]	94	60–85	retrospective study,	orthopedic surgery	paracoxib vs. control	MoCA score higher in the paracoxib group; TNF-α, IL-6, IL-1β lower, IL-10, MCP-1 higher in the paracoxib group; **implication**: parecoxib can notably inhibit postoperative inflammatory cytokines, improve neurological function, and reduce the occurrence of POCD
Huo QF et al. [166]	397	65–80	RCT	total hip arthroplasty	DEX vs. UTI vs. DEX-UTI	POCD incidence lower in DEX-UTI group; IL-6 lower in DEX-UTI group; **implication**: combination of UTI and DEX has a protective effect on cognitive function and reduces emergence agitation and inflammation
Ye C et al. [167]	218	65–90	RCT	thoracolumbar compression fracture surgery	DEX vs. control	POD incidence lower in DEX group; IL-6, TNF-α lower in DEX group, IL-1 no significance; **implication**: DEX reduces the incidence of POD and lowers pro-inflammatory cytokine levels, thereby improving cognitive function
Ma X et al. [168]	108	18–70	RCT	resection of gastrointestinal tumor resection	narcotrend vs. physician experience	POCD incidence lower in the narcotrend group; IL-1β, TNF-α lower in the narcotrend group; **implication**: narcotrend monitoring of anesthetic depth reduces the degree of POCD

AAI, auditory evoked potential index; BIS, bispectral index; CSF, cerebrospinal fluid; DEX, dexmedetomidine; IL, interleukin; IFN-γ, interferon gamma; MCP-1, monocyte chemoattractant protein-1; NMB, neuromuscular blockade; MMSE, Mini-Mental State Exam; MoCA, Montreal Cognitive Assessment; S100B, S100 calcium binding protein B; Sevo, sevoflurane; TNF-α, tumor necrosis factor alpha; PRE, preoperative; POST, postoperative; PRE–POST change, preoperative–postoperative difference; POCD, postoperative cognitive dysfunction; POD, postoperative delirium; RCT, randomized controlled trial; TIVA, total intravenous anesthesia; UTI, ulinastatin; Qmci-TW, Taiwan version of quick mild cognitive impairment screen.

Jiang et al. [126] investigated 142 patients aged 18–80 years undergoing esophageal cancer surgery with or without the addition of epidural block to general anesthesia. They demonstrated that combining general and epidural anesthesia reduced the incidence of POCD by mitigating the inflammatory response. Among RA-related studies, the findings of Feng et al. [122] are particularly noteworthy. They evaluated the effects of different fascia iliaca compartment block (FICB) approaches on postoperative outcomes, including POCD, in 60 patients aged 18–80 years undergoing total hip arthroplasty under general anesthesia with sevoflurane. The FICB was performed using two methods: the “inside” and “outside” approaches. The inside FICB approach provided superior anesthetic effects, improved postoperative analgesia, reduced reliance on postoperative analgesics, and resulted in a lower incidence of POCD. The higher levels of IL-1β and IL-6 observed in the outside approach were identified as factors contributing to the increased POCD incidence in this group.

### 4.2. Impact of DEX on PNDs from the Perspective of Neuroinflammation and Cytokines

A recent meta-analysis indicated that the use of midazolam, propofol, desflurane, and sevoflurane is associated with a higher incidence of delirium compared to DEX. DEX, an α2 adrenoceptor agonist, was first approved for clinical use by the FDA in 1999, primarily for sedation. In animal models of systemic inflammatory responses and surgical injury, DEX has demonstrated the ability to mitigate neuroinflammation and neuroapoptosis. A literature review indicated that DEX exerts its neuroprotective effects primarily through the upregulation of α2 adrenoreceptors. Preclinical studies have indicated that DEX significantly reduces neuroinflammation and neurodegeneration following neurological injury by decreasing hippocampal expression of IL-1β, IL-6, and TNF-α, as well as reducing the activation of astrocytes and microglia. DEX improves neuroinflammatory responses by inhibiting inflammatory mediators, regulating apoptotic signaling pathways, and reducing the production of oxygen-free radicals [169,170].

In an early study on DEX, Li Y et al. [130] demonstrated that its combined use with propofol-based general anesthesia reduced the incidence of POCD. This effect was attributed to a reduction in the inflammatory response, as evidenced by decreased levels of IL-1β and IL-6. Similarly, a recent study by Ye C et al. [167] reported that the combined use of DEX and propofol-based general anesthesia decreased the incidence of POD. Additionally, they observed reduced IL-6 and TNF-α levels, concluding that these reductions indicate that a neuroinflammatory response is the underlying mechanism. Meanwhile, Kim JA et al. [141] conducted a study involving 143 patients undergoing thoracoscopic lung resection surgery to investigate the effects of adding DEX to sevoflurane anesthesia on emergence agitation and POD. They reported that while DEX reduced emergence agitation, it did not decrease the incidence of POD. Additionally, they observed higher IL-6/IL-10 and IL-8/IL-10 ratios in the group receiving DEX, indicating a pro-inflammatory cytokine balance in the DEX group. Furthermore, norepinephrine and epinephrine levels were lower in the DEX group, leading them to conclude that the reduction in emergence agitation was due to the effect of catecholamines rather than anti-inflammatory action.

Several studies have explored the effects of DEX administration methods and dosage on PNDs. Lee C et al. [139] investigated the relationship between the timing and dosage of DEX administration and POD incidence. They found that preoperative, prophylactic continuous infusion of DEX significantly reduced the incidence of POD in elderly patients for up to 5 days following laparoscopic major non-cardiac surgery. Furthermore, prophylactic DEX infusion, regardless of timing or dosage, decreased the duration of POD. Regarding inflammatory cytokines, their study showed that IL-6 levels were significantly lower in patients who received higher doses of DEX with bolus and continuous infusion during surgery compared to those who received lower doses via bolus injection at the end of surgery, correlating with a lower incidence of POD.

Tang Y et al. [153] examined the effect of DEX dosage on PNDs in elderly patients undergoing hepatic lobectomy. They concluded that an intraoperative DEX infusion at a dose of 0.3 or 0.6 μg/kg/h reduces the incidence of POCD and POD. This protective mechanism likely involves the downregulation of TNF-α and IL-1β and the upregulation of IL-10 expression, restoring the balance between pro-inflammatory and anti-inflammatory responses.

### 4.3. Potential Therapeutic Approaches Targeting Cytokines

Various studies have explored the relationship between anesthesia and PNDs from the perspectives of neuroinflammation and cytokines. Research on the depth of anesthesia has shown that adjusting the depth using anesthesia depth monitoring equipment [129,168] and maintaining deeper anesthesia [140] can reduce the incidence of POCD. Deeper levels of anesthesia were consistently associated with decreased POCD occurrence.

Studies investigating the effect of ulinastatin (UTI) administration on PNDs have also been reported [128,134,166]. UTI, derived from human urine, is known to inhibit enzyme activity, stabilize lysosomal membranes, and effectively reduce systemic inflammatory responses. It achieves this by directly suppressing the activation of neutrophils and monocyte-macrophages, as well as by capturing LPS and binding to LPS receptors, thereby inhibiting LPS-induced systemic inflammatory responses. UTI has been shown to reduce the incidence of POCD and lower the levels of IL-6 and S100B compared to controls, indicating that UTI alleviates neuroinflammatory responses.

Remote ischemic preconditioning, a method involving repeated temporary restriction of blood flow to a limb, has demonstrated promising neuroprotective effects. Studies in rats have demonstrated that remote ischemic preconditioning protects against cerebral ischemia by modulating peripheral immune responses [171]. However, clinical trials on this technique are limited. He Z et al. [136] investigated the effects of ischemic preconditioning on cognitive function in 90 patients aged 65–75 years undergoing laparoscopic colorectal cancer surgery. They found that remote ischemic preconditioning improved early postoperative cognitive function and reduced the serum levels of S100B, IL-1β, and TNF-α. These improvements in cognitive function were attributed to the inhibition of the inflammatory response triggered by surgery. This method offers a non-pharmacological approach with significant potential for clinical application as a less invasive alternative to cerebral preconditioning.

## 5. Future Directions

The studies summarized in Table 2 demonstrate inconsistencies, particularly in cytokine-related outcomes, which vary across investigations. Unfortunately, despite ongoing research into various anesthetic agents and methods aimed at reducing PNDs by mitigating neuroinflammatory responses, a definitive conclusion has yet to be reached. This is due to the limited number of studies available for meta-analysis, as well as significant differences across studies in terms of patient age, types of surgeries, and anesthetic techniques used. There is still insufficient evidence to draw definitive conclusions about the relationship between PNDs and neuroinflammation, as well as the anesthetic agents and methods to reduce its occurrence. Additionally, this review did not conduct a comprehensive meta-analysis by focusing solely on the cytokine pathway. This is a clear limitation, as it restricts the generalizability of the findings. Further preclinical and well-designed clinical studies are needed to better understand the precise mechanisms linking surgery, neuroinflammation, and the development of neurological disorders. Furthermore, research should focus on evaluating the efficacy and safety of therapeutic agents in mitigating these post-surgical complications. To achieve this, the authors suggest that large-scale multicenter studies should be actively conducted, involving a unified age group of participants and standardized surgical procedures, as well as consistent anesthetic methods. Only through such studies will it be possible to draw robust conclusions regarding the mechanisms and treatments of PNDs and the impacts of anesthesia, supported by meta-analysis.

## 6. Conclusions

In conclusion, the exact pathogenesis of PNDs remains unclear. Recent human and animal models have indicated that neuroinflammation triggered by surgery or anesthesia plays a significant role in PND onset and progression. The inflammatory response induced by surgery and anesthesia has been shown to compromise BBB integrity, leading to proinflammatory cytokine infiltration, microglia activation, and the induction of neuroinflammatory responses. These processes ultimately contribute to the development of PNDs. Several studies have sought to reduce PNDs by modifying anesthetic agents and techniques. Unfortunately, the evidence supporting these interventions remains limited. Therefore, further research involving a standardized participant age group, consistent surgical procedures, and uniform anesthetic methods is required to achieve more reliable conclusions and identify effective strategies for mitigating PNDs.

## Data Availability

Not applicable.
